# P-2184. Antiviral Use among Immigrants with Chronic Hepatitis B (HBV) in Metropolitan-DC

**DOI:** 10.1093/ofid/ofae631.2338

**Published:** 2025-01-29

**Authors:** Christine Lin, Huong Dang, Lydia Tang

**Affiliations:** University of Maryland School of Medicine, Baltimore, Maryland; Dang Huong MD, Falls Church, Virginia; Institute of Human Virology, University of Maryland school of medicine, Baltimore, Maryland

## Abstract

**Background:**

Globally, 296 million people are infected with chronic hepatitis B (HBV) and at risk for cirrhosis and hepatocellular carcinoma. In the Metropolitan-DC area, chronic hepatitis B infection (HBV) is 10 times more common in Asian, African, and Latin American communities (5% prevalence) than non-immigrants communities. Therefore, HBV-related liver disease, including cancer, is a major health disparity for immigrants. Antivirals reduce cancer risk but require long-term adherence. Immigrants face barriers to HBV antiviral adherence, including language, high cost of antivirals, and establishing care for HBV management. We examine antiviral use in a cohort of people with HBV.

**Methods:**

HOPE is a natural history study at the University of Maryland, Baltimore. HOPE enrolls adults with HBV and includes TEMUL, a sub-study providing free tenofovir alafenamide (TAF) for 2 years. After completing 2 years of TEMUL, participants are transitioned to receiving antivirals through their non-study healthcare providers. We describe antiviral use while on TEMUL and at a 2-year follow-up.

**Results:**

From May 2017 to March 2024, 92 people received TAF through TEMUL. Eighty-four were foreign-born (78 born in Asia and 6 born in Africa). Most (67%) had mild liver fibrosis at baseline. Eleven were withdrawn (3 lost to follow-up, 3 moved out of state, 5 voluntarily withdrew). Eighty-one completed TEMUL with sustained adherence to TAF. Fifty-eight completed a 2-year follow-up. At follow-up, 36 of 58 (62%) remained on TAF without interruption. Twelve (21%) switched antivirals because of insurance coverage. This caused lapses in antiviral use ranging from 1 month to 2 years in 5 (9%). Ten (17%) stopped antivirals due to loss of health insurance, high copay, and inability to follow-up with a healthcare provider. All 10 were foreign-born, Asian, and non-English speaking. Of these 10, 9 were viremic at follow-up and resumption of antivirals was clinically indicated in all.

**Conclusion:**

Language and inadequate health insurance coverage hinder antiviral adherence in immigrants with HBV. Foreign-born, non-English speaking patients may be especially vulnerable to lapses in antiviral use. Interventions to improve access to antivirals tailored to this patient group are needed.

**Disclosures:**

Lydia Tang, MBChB, Arbutus Biopharma: Grant/Research Support|Gilead Sciences: Grant/Research Support

